# Use of a Tri-Axial Accelerometer Can Reliably Detect Play Behaviour in Newborn Calves

**DOI:** 10.3390/ani10071137

**Published:** 2020-07-05

**Authors:** Nicola Gladden, Erin Cuthbert, Kathryn Ellis, Dorothy McKeegan

**Affiliations:** 1 Scottish Centre for Production Animal Health and Food Safety, University of Glasgow School of Veterinary Medicine, Bearsden Road, Glasgow G61 1QH, UK; Kathryn.Ellis@glasgow.ac.uk; 2 Institute of Biodiversity, Animal Health & Comparative Medicine, University of Glasgow Garscube Estate, Bearsden Road, Glasgow G61 1QH, UK; erin.cuthbert@gov.bc.ca (E.C.); Dorothy.McKeegan@glasgow.ac.uk (D.M.)

**Keywords:** welfare, calf behaviour, accelerometer, play, positive welfare state

## Abstract

**Simple Summary:**

Traditionally, health and production measures have been used to assess farm animal welfare, but these do not encompass all aspects of welfare. In recent years, the concept of “positive animal welfare” has been gaining momentum, in line with the notion that a good animal life prevents negative experiences and also promotes positive experiences. Play behaviour is considered to be a good indicator of positive animal welfare. Accelerometers (movement sensors) worn by animals can be used to monitor activity as a proxy for different behaviours in a much less time-consuming manner than traditional behavioural observations. In this study, we assessed whether a commercially available leg-mounted accelerometer could reliably identify play behaviour in newborn dairy calves. Our results showed that accelerometer technology can be used to identify the amount of play behaviour exhibited by newborn calves in the first 48 h of life, and we discuss its potential for use in the assessment of the welfare of newborn calves in the future.

**Abstract:**

Traditionally, the welfare assessment of farm animals has focused on health and production outcomes. Positive welfare is, however, not merely the absence of negative welfare and is an important part of a life worth living. Play behaviour is widely considered to be an indicator of positive emotions because it is a “luxury” behaviour. Direct visual observation is considered the most accurate method of behavioural analysis, but it is time consuming and laborious. There is increasing interest in the use of remote monitoring technology to quantify behaviour. We compared the data output (“motion index” (MI)) from a commercially available tri-axial accelerometer fitted to newborn dairy calves to video footage of the same calves, with a focus on play behaviour. The motion index values over 48 h were positively correlated with both the duration of play behaviour and the number of play bouts. The motion index threshold in each sample interval with the optimal sensitivity and specificity for the identification of play behaviour was MI ≥ 2.5 at a 1 min resolution (sensitivity (Se) = 98.0%; specificity (Sp) = 92.9%) and MI ≥ 24.5 at a 15 min resolution (Se = 98.0%; Sp = 89.9%), but these values consistently overestimated the overall proportion of sample intervals in which play was observed. The MI that best reflected the results obtained from visual one-zero sampling was MI ≥ 23 for 1 min intervals and MI ≥ 62 for 15 min intervals—this may therefore be the basis of a more conservative approach to the identification of play behaviour from accelerometer-generated data. Our results indicate that accelerometer-generated data can usefully indicate the amount of play behaviour shown by newborn calves for up to 48 h, providing an efficient method for identifying this important parameter in future work.

## 1. Introduction

There is increasing interest in the welfare of farmed animals among both consumers [1] and scientists [2]. Welfare assessments are commonly included in farm quality assurance schemes across the farming industry [3,4,5,6] but typically employ welfare indicators such as the presence or absence of disease and the availability (or lack thereof) of resources (e.g., space allowances) [3,7,8]. Whilst these factors are straightforward to measure, they tend towards the assessment of the absence of a negative welfare state and do not consider the presence (or absence) of positive welfare states. In recent years, the concept of identifying and assessing the presence of good welfare conditions and positive welfare states has been gaining traction [9]. Positive animal welfare encompasses the concepts of positive emotions, positive affective engagement, happiness and good quality of life [9] and, as such, is potentially more difficult to quantify than traditional welfare measures such as husbandry standards and health outcomes.

Behavioural analysis has shown promise as a method of assessing positive welfare [10,11] and is recommended for inclusion in on-farm welfare assessment protocols [12]. Detailed behavioural analysis is time consuming and labour intensive [13], which means its routine inclusion in on-farm welfare assessment protocols is rarely feasible [11]. Continuous visual observation provides an exact record of the behaviours observed, but it is only practical for behavioural analysis over short time periods [14]. Other methods of behavioural analysis such as instantaneous sampling and one-zero sampling techniques have been devised to improve the feasibility of analysing behaviour over longer time periods and also allow larger numbers of behavioural categories to be measured at the same time [14,15]. Time budgets constructed using these methods correlate well with continuous observations for common and long-duration behaviours [16]; however, the time budgets constructed from these methods often underestimate the duration of time engaged in short-duration or infrequent behaviours [17], and selecting an appropriate sampling interval in studies where several different behaviours are being observed can be difficult [18]. Recent technological advances have allowed animal activity to be recorded automatically using animal-mounted activity monitors [19,20]. Activity can be monitored over long periods of time, and data can be downloaded for detailed analysis at any point in time at which it is required, conferring advantages over the visual observation of animals either pen-side or with video recordings. The challenge, therefore, is to devise remote activity-monitoring technology that maintains an accurate correlation with continuous visual observations, even for rare or short-duration behaviours [19,21,22]. Whilst remote monitoring devices typically record measurements at set sampling intervals [21,23]—analogous to instantaneous sampling—the sampling intervals are much shorter than those that would be practical or even possible for visual observations (sometimes fractions of a second) and, consequently, are a close approximation for continuous visual observations. A wide range of different animal-mounted sensors are now available for the remote monitoring of animal health and behaviour [24], and leg-mounted tri-axial accelerometers are commonly used in behavioural studies [19]. This type of accelerometer contains a piezoelectric sensor that generates a voltage signal in response to any change in velocity experienced in three planes and produces outputs representative of three-dimensional movement [19].

Accelerometer technology has been utilised to monitor different behaviours in a wide variety of wild and domestic species including cattle [19,25,26,27,28,29]. Accelerometers have been evaluated as tools for identifying many different types of bovine behaviour including lying behaviours [30,31,32,33,34,35], locomotion [34,36,37], feeding/drinking behaviours [35,38,39,40] and play behaviour [41,42]. Whilst accelerometer generated data have shown good correlation with visual observations for standing and lying behaviours in adult cows [21,32,35,43,44] and lying behaviours in calves [30,45], the reported correlation between accelerometer measurements and locomotor activity in calves is inconsistent. For example, Luu et al. [41] reported a good correlation between the number of acceleration peaks and the duration of time engaged in running, jumping/kicking and walking (*r* = 0.96, 0.86 and 0.75, respectively), whereas Trénel et al. [33] reported a low sensitivity (raw data sensitivity = 0.15; filtered data sensitivity = 0.22) for identifying movement in calves. Some studies have evaluated the use of accelerometers to identify play behaviours in calves [41,42,46,47], but these evaluate behaviour in calves aged four weeks or older [41,42,46], evaluate play behaviour in arena tests [41,46,47] or apply data manipulation methods to the raw accelerometer data [41]. No studies have evaluated the use of accelerometers to identify play behaviour in neonatal calves aged up to 48 h old in their home pen using the raw accelerometer data that would be available to farmers, welfare auditors and veterinarians.

Play behaviour is observed in almost all species’ young and is widely considered to be an indicator of good welfare that occurs when an animal’s basic needs (e.g., nutrition) are fulfilled [48,49,50]. Play behaviour is not considered to be essential for survival, and animals typically do not expend energy expressing play behaviour when resources are limited or welfare is compromised [48]; play is therefore considered to be a “luxury behaviour” that is exhibited when animals are in a positive welfare state [51]. Play is also thought to be an indicator of positive emotions in animals [50,52]; however, play behaviour can be time consuming and laborious to assess as it occurs spontaneously, infrequently and over short durations [42]. Play cannot be predicted, and long periods of observation are required to accurately determine the duration and number of play bouts, making the assessment of play behaviour impractical for inclusion into on-farm welfare assessments. Accelerometer technology can potentially mitigate these limitations, and, as such, the use of accelerometers to identify play behaviour for the purposes of the assessment of positive states is of interest. Größbacher et al. [42] assessed locomotor play in group-housed calves aged four and eight weeks old and found that the Hobo Pendant G Acceleration Data Logger (Onset Computer Corporation, Bourne, MA, USA) correctly identified 79% of the sampling periods in which play occurred but consistently overestimated play behaviour, and a correction factor had to be applied to enable the accelerometer derived data to reflect visual one-zero sampling. They concluded that, although this accelerometer provided a good approximation of spontaneous locomotor behaviour in calves, the sensor did not have a high enough recording frequency (1 Hz) for the accurate measurement of play behaviour [42].

The synchronisation of observed behavioural patterns with device-generated data is considered best practice for validating remote monitoring devices as tools for recording behaviour [19]. The IceTag accelerometer (IceRobotics, South Queensferry, UK) was chosen for use in this study as it is a small device (measuring 66.0 mm × 55.0 mm × 27.0 mm), weighing only 117 g, and as such, it was unlikely to cause disruption to the calves’ normal behaviour. It also has a high frequency of data collection of 16 Hz (i.e., 16 samples are measured every second) and the data are presented in intervals as short as one second, ideal for potentially capturing short duration behaviours such as play in calves.

The objectives of this study were to determine whether IceTag-generated motion index (MI) data had the potential to identify play behaviour in very young dairy calves (up to 48 h old). IceTag-generated MI data were compared to detailed focal observations of calf behaviour, and different analytical approaches were used to define the optimal method of utilising MI for identifying play behaviour. Initial work was undertaken to investigate whether the number of locomotor play bouts or the duration of locomotor play in the first 48 h of life were correlated with the cumulative IceTag-generated MI data. Although valuable information was obtained from this correlation analysis, it is a crude measure of behaviour; hence, more detailed analyses were subsequently performed. Firstly, an epidemiological approach was employed to calculate the sensitivity and specificity of selected MI cut points (thresholds) for detecting play behaviour with 1 min and 15 min sampling intervals. Sensitivity and specificity are test performance characteristics indicating the ability of a diagnostic test (in this case, MI values) to correctly detect the presence or absence of a condition (in this case, locomotor play) [53]. This approach provides detailed information about the ability of MI to detect the presence or absence of play behaviour in each sample interval; however, it does not provide information on the amount of sample intervals during which play occurs, or information about behavioural patterns over time. The motion index is a single figure generated by the IceTag for selected sample intervals; therefore, it can only detect whether play was present or absent in each sample interval—this is analogous to one-zero behavioural sampling [14]. One-zero sampling records whether (or not) a behaviour was observed in a sample interval selected by the investigator and can be used when the presence or absence of a behaviour is the point of interest [54]. Thus, our final analysis compared selected MI thresholds to the results obtained from the visual one-zero behavioural analysis of video footage, aiming to establish a practical method of analysing IceTag-generated data that might have potential for future use in the on-farm welfare assessment of neonatal calves up to 48 h old.

## 2. Materials and Methods

### 2.1. Calf Recruitment and Data Collection

An 800-cow Holstein dairy herd in Scotland, UK was recruited to the study. Calf management was as described previously [55,56]; briefly, calves are removed from the dam soon after birth (≤8 h) and housed in age-matched groups of four to six. All the calves are weighed and fed 4.5 L of colostrum within the first 4 h of life. The management of the calves recruited to the study was the same as that of all the other calves on the farm, with the exception of them being fitted with an IceTag accelerometer shortly after birth and being marked with agricultural marker spray to aid video identification. All the calves that were expected to remain on the farm for at least 48 h after birth were eligible for recruitment; calves were recruited in accordance with IceTag availability.

Calf behaviour was continuously filmed using closed circuit television (CCTV) cameras (Sony CCD, Vari-focal, 700 TV L, Sony, Minato, Japan) as described previously [55], and the video footage was stored on digital video recorders (DVR) (Guardian II+DVR 8 Channel, Digital Direct Security, Huntingdon, UK) on the farm. The required footage was regularly backed up onto an external hard drive (Seagate 1TB portable external hard drive, Seagate Technology LLC, Cupertino, CA, USA) for long-term storage and to facilitate analysis.

An IceTag accelerometer (IceTag, IceRobotics, South Queensferry, UK) was placed on the lateral aspect of one hindlimb in accordance with the manufacturer’s instructions; the IceTag was held in place using a cohesive bandage (Cattle Wrap, Andover, MA, USA). For calf comfort, a fabric sock was also used to cushion the IceTag in a method established during a pilot feasibility study (Ord, unpublished). IceTags were activated using an IceReader wireless download device (IceReader, IceRobotics, South Queensferry, UK) together with the IceRobotics IceManager software (IceManager, IceRobotics, South Queensferry, UK) [57]. The IceTags were removed from each calf after the 48 h recording period was complete.

For the purposes of identification on the video footage, the calves were marked using agricultural marker spray, and each calf was photographed to further aid and confirm identification; for the purposes of analysis, the calves were identified by the last four digits of their official UK identification number. The date and time of birth and the animal identification number were recorded by the farm staff. The calf birthweight, the number of the pen the calf was moved to and the time at which each calf was moved into the pen were all recorded by the researcher (E.C.). The colour of the cohesive bandage, the IceTag identification number and time of the IceTag activation were recorded at the beginning of the 48 h observation period by the researcher (E.C.).

Data were collected in two sampling periods: 8 May to 27 May 2019 and 18 July to 2 August 2019. Ethical approval for the study was obtained from the University of Glasgow School of Veterinary Medicine Research Ethics Committee (Ref: EA13/19).

### 2.2. Extraction of IceTag Accelerometer Data

The IceManager software [57] generates a report from the downloaded raw IceTag data that can be exported in comma-separated values (CSV) format for further analysis. The output resolution (sample interval) is dictated by the software; the available options for selection are 1 s, 1 min, 15 min, 1 h, 2 h, 1 d and 1 week.

The motion index (MI) is a proprietary metric calculated by the IceTag accelerometer for each output resolution. It is calculated from the average vector sum acceleration over all three dimensions, thus producing a total value that is expressed as a single figure. The motion index is affected by both the duration of activity and the vigour of leg movement; it is therefore an indicator of overall activity.

### 2.3. Correlation between Motion Index and Behavioural Observations

#### 2.3.1. Behavioural Analysis

The observation and recording of behaviours from video footage was performed using the freely available open-source event-logging program Behavioral Observation Research Interactive Software (BORIS) (BORIS v.7, Torino, Italy) [58]. Behavioural observations in BORIS were synchronised to the time stamp on each video file with an accuracy of 1 s. An ethogram for calf behaviour based on that previously described [49,55] was defined for use (Table 1). For the purposes of analysis, behaviours were grouped into three primary categories according to how they might be differentiated by the IceTag accelerometer output: “resting” (i.e., lying behaviours), “active” (all non-lying behaviours except for play) and “play”. The recording of play bouts began at the onset of play behaviour and ended when play was discontinued for more than one second. For completeness, specific play behaviours (e.g., head shaking) were recorded at the time of occurrence within play bouts; however, as the IceTag accelerometer was not expected to pick up detailed play behaviours that did not involve leg movement, these were excluded from this and further analyses, and only locomotor play was included (hereafter referred to as “play”). Calves that could not be observed were recorded as “out of view”.

Continuous visual observations were completed for 12 calves. Data were analysed for the complete 48 h time period and also separately for each 24 h observation period (0 to 24 h, 12 to 24 h, 24 to 24 h and 36 to 48 h). This approach was chosen because we have previously demonstrated that the proportion of the time budget that calves of this age engage in play behaviour is affected by time over the first 48 h of life [55]. The total MI was correlated with the duration(s) and counts of bouts of active behaviours excluding play (i.e., standing and walking behaviours combined), active behaviours including play (i.e., standing, walking and locomotor play combined) and play alone. This allowed us to determine whether correlations between the MI and play behaviour were related to locomotor play specifically or were due to differences in general activity.

Complete IceTag datasets were only available for all 12 calves for the 0 to 24 h observation period. For the 12 to 24 h observation period, complete IceTag data were available for 11 calves (one calf was removed from the study due to illness), and for the 24 to 24 h and 36 to 48 h observation period, complete IceTag data were available for ten and nine calves, respectively (two further calves had missing data due to sensor malfunction). All calves were included in the combined 48 h analysis, as the number of hours of behavioural data available were matched to the number of hours of IceTag data available, and therefore, missing data were accounted for in the analysis.

#### 2.3.2. Statistical Analysis

The duration(s) and number of bouts of each behaviour (calculated in BORIS) and the IceTag-generated MI data for each corresponding observation period were collated in an Excel spreadsheet (Microsoft Excel v.1908, Microsoft, Redmond, WA, USA) and exported to Minitab (Minitab v.18, Minitab LLC, State College, PA, USA) for analysis. The data were examined for normality by the visual appraisal of histograms and Anderson–Darling normality analysis.

The cumulative MI generated by the IceTag accelerometer for each 24 h sample period was correlated to the duration(s) and number of bouts of each behaviour; time periods were matched with an accuracy of 1 s. Prior to analysis, all data were examined to verify that the assumptions of each correlation analysis were met. A Pearson’s correlation coefficient (*r*) was calculated for pairs of data where at least one variable was normally distributed. If neither dataset was normally distributed, a Spearman’s rank correlation coefficient (*r_s_*) was calculated. The threshold for significance was set at *p* < 0.05.

### 2.4. Calculation of Sensitivity and Specificity and Comparison with One-Zero Sampling

#### 2.4.1. Behavioural Analysis

The detection of play behaviour in different sampling intervals was the focus of this analysis; therefore, a more simplified ethogram was defined for use (Table 2). The behavioural descriptions defined in this ethogram were selected to reflect the patterns of behaviour captured by the IceTag accelerometer; i.e., behaviours that involve leg movement. All the behaviours recorded were exclusive of each other with the exception of “side stepping”; this behaviour could occur concurrently with standing and was recorded because it is a nested behaviour that could potentially be captured by the IceTag whilst the calf was standing. The observation and recording of behaviours was performed using BORIS (BORIS v.7, Torino, Italy) with the synchronisation of video footage and observation time stamps as previously described.

Sampling intervals of 1 min and 15 min duration were chosen for analysis based on pilot work. The sampling intervals were dictated by the IceTag software, and data recording started at the start of the first complete 15 min interval available. Observations were recorded for the duration of one hour every fourth hour of the total available observation period; the video footage of each calf was analysed using one-zero recording [14]. For each calf, both the observational data recorded in BORIS and IceTag-generated data were exported into the same Excel spreadsheet and synchronised in binary format, recorded as either play observed (Y) or play not observed (N) in each sampling interval.

For the purposes of analysis, ten calves were selected based on the completeness of the available data. Data obtained from all calves were used to determine the MI with optimum sensitivity and specificity for detecting play behaviour in 1 min and 15 min intervals (5938 and 396 sampling points, respectively). Subsequently, the data were divided into two equal groups, and visual observations recorded in one-zero sampling format were compared to the motion index. The data obtained from the first group of calves were used to determine the MI threshold that best reflected visual one-zero observations in 1 min and 15 min intervals (3501 and 234 sampling points, respectively). This metric was then applied to the data obtained from the second group of calves to assess the repeatability of the findings for a new dataset (2437 1 min sample points and 162 15 min sample points).

#### 2.4.2. Statistical Analysis

##### Calculation of Sensitivity and Specificity of MI to Detect Play Behaviour

For the selected MI cut points (a range of 3 to 60 for 1 min intervals and range of 20 to 300 for 15 min intervals), data were formatted in 2 × 2 contingency tables for the calculation of the sensitivity (Se) and specificity (Sp) of each MI cut point for detecting the occurrence of play [53] and the balanced accuracy for each cut point. The sensitivity of each selected MI cut point was calculated using the formula:

Se = (number of true positive results)/(number of true positive + false negative results)

The specificity of each selected MI cut point was calculated using the formula:

Sp = (number of true negative results)/(number of true negative + false positive results)

The balanced accuracy was calculated for each cut point using the formula [59]:

(Sensitivity + specificity)/2

A true positive result was defined as a result where play behaviour was observed to be occurring in a sample interval where the MI equaled or exceeded the selected cut point. A false positive result was defined as a result where play behaviour was not observed to be occurring in a sample interval where the MI equaled or exceeded the selected cut point. A true negative result was defined as a result where play behaviour was not observed to be occurring in a sample interval where the MI was equal to or less than the selected cut point. A false negative result was defined as a result where play behaviour was observed to be occurring in a sample interval where the MI was equal to or less than the selected cut point.

The single MI cut point for detecting play behaviour was optimised using Classification and Regression Tree (CART) analysis [60,61]. All the data were exported into Minitab (Minitab v.19, Minitab LLC, State College, PA, USA), and Classification and Regression Tree analysis was performed for both the 1 min and 15 min sampling intervals using the Gini node splitting method. As the aim of this study was to establish whether a single MI cut point had potential for detecting locomotor play behaviour, the simplest (two node) tree was selected for each sample interval. Validation was performed for each sample interval using 10-fold cross validation [62,63].

##### Comparison between Motion Index and Visual One-Zero Sampling

The motion index for the same selected cut points as used to calculate sensitivity and specificity was compared to the results of the visual one-zero sampling. Using one dataset, a one-zero sampling score was calculated for the visual observations using the formula [14]:

Number of intervals where play occurred/total number of sampling intervals

An equivalent score was calculated for selected MI cut points using the formula:

Number of intervals where MI ≥ selected cut point/total number of sampling intervals

The MI threshold that best reflected visual one-zero sampling was defined by comparing the visual one-zero sampling score (the reference measurement) with the equivalent score calculated for each selected MI. An equivalent one-zero sampling score was calculated for different MI thresholds until the MI that delivered the result closest to the visual one-zero sampling score was identified; this was the optimal MI threshold for detecting play behaviour when compared to visual one-zero sampling. This MI was validated by applying it to the second dataset; this is a similar principle to the test/train validation technique.

## 3. Results

### 3.1. Correlation between Motion Index and Behavioural Observations

The motion index was positively correlated with both the total number of play bouts and the total duration of play for the complete 48 h observation period as well as for the 12 to 24 h, 24 to 24 h and 36 to 48 h observation periods (Table 3). The motion index was also positively correlated with the number of bouts of active behaviour inclusive of play in the 12 to 24 h, 24 to 24 h and 36 to 48 h observation periods. A significant positive correlation between the MI and the duration of active behaviour inclusive of play was also identified in the 24 to 24 h and 36 to 48 h observation periods, but not the 12 to 24 h observation period (Table 3). The motion index was positively correlated with the total number of bouts of active behaviour (excluding play) in the 24 to 36 h observation period only and was positively correlated with the duration of active behaviour (excluding play) in the 24 to 36 h and 36 to 48 h observation periods (Table 3).

There was no correlation between the MI and the total duration of lying behaviour in any observation period; however, the total number of lying bouts was positively correlated with the motion index in the 24 to 36 h observation period and also for the total combined 48 h observation period. No behaviours were correlated with the MI in the 0 to 12 h observation period.

### 3.2. Sensitivity, Specificity and Comparison with One-Zero Sampling

#### 3.2.1. Sensitivity and Specificity

Of the selected MI cut points analysed, the maximum specificity was associated with a MI cut point of ≥300 for a 15 min resolution and ≥60 for a 1 min resolution (Sp = 100% and 99.8%, respectively); however, the sensitivity was only 19.6% and 21.7%, respectively, for these cut points, and the balanced accuracy was moderate (Table 4 and Table 5). The maximum sensitivity was associated with MI cut points of both ≥20 and ≥25 (Se = 98.0%) for a 15 min resolution and ≥3 (Se = 98.0%) for a 1 min resolution; the specificity for these cut points was 87.8% (MI ≥ 20) and 89.9% (MI ≥ 25) for the 15 min resolution and 92.9% (MI ≥ 3) for the 1 min resolution (Table 4 and Table 5).

**Figure 1:**
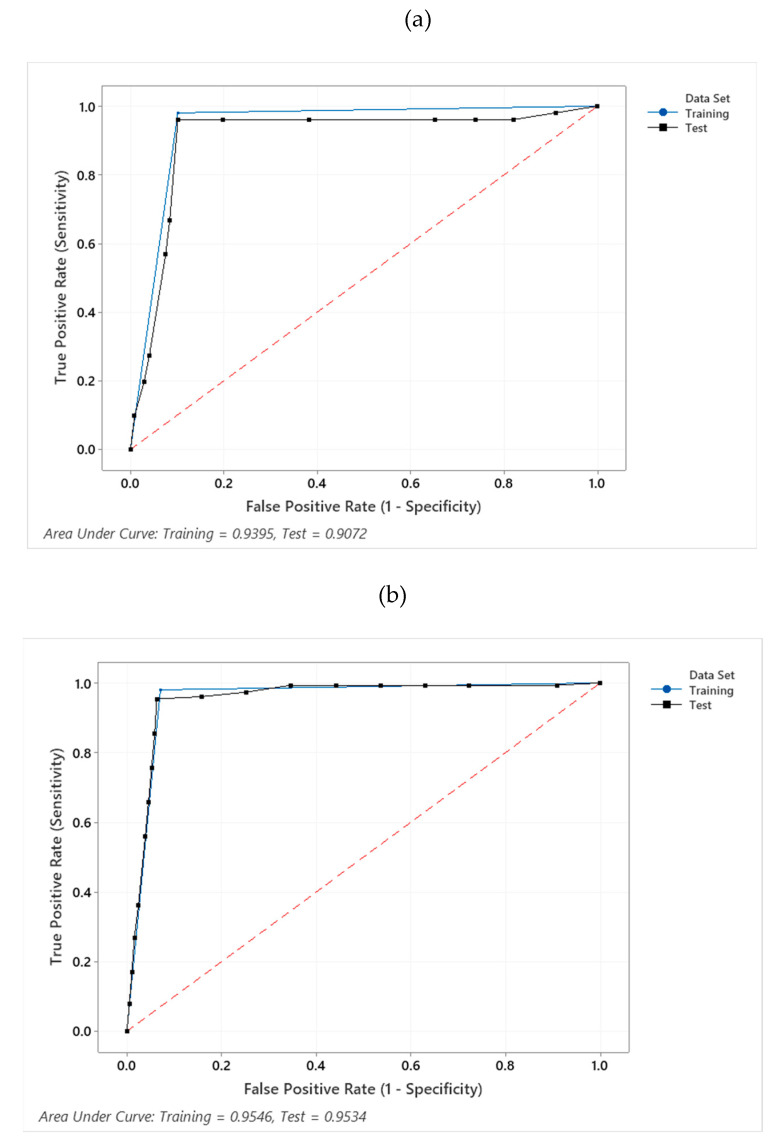
(**a**) Receiver operating characteristic (ROC) curve demonstrating the sensitivity and specificity of MI ≥ 24.5 for detecting play behaviour in 15 min sample intervals as calculated from a training dataset (blue line) and when applied to a test dataset (black line) using 10-fold cross validation. (**b**) Receiver operating characteristic (ROC) curve demonstrating the sensitivity and specificity of MI ≥ 2.5 for detecting play behaviour in 1 min sample intervals as calculated from a training dataset (blue line) and when applied to a test dataset (black line) using 10-fold cross validation.

Classification and Regression Tree analysis indicated that the optimum motion index cut point for detecting the occurrence of play behaviour in 15 min sampling intervals was MI ≥ 24.5 (Se = 98.0%; Sp = 89.9%), which performed well when applied to the validation set using 10-fold cross validation (Se = 96.1%; Sp = 89.9%) (Figure 1a). The optimum motion index cut point for detecting the occurrence of play behaviour in 1 min sampling intervals was MI ≥ 2.5 (Se = 98.0%; Sp = 92.9%); this value also performed well when validated (Se = 95.4%; Sp = 93.7%) (Figure 1b). However, both of these cut points overestimated the proportion of sampling intervals in which play occurred (Table 6).

#### 3.2.2. Comparison between Motion Index and Visual One-Zero Sampling

The MI thresholds that generated the most accurate one-zero equivalent sampling scores compared to the one-zero sampling score produced by visual observations were MI ≥ 23 for 1 min sampling intervals and MI ≥ 62 for 15 min intervals (Table 6). These thresholds were highly repeatable when applied to the second dataset: a difference of less than 0.1 was observed between the one-zero sampling scores produced by visual observations and equivalent scores produced by using the MI data for both 1 min and 15 min sampling intervals (Table 6).

## 4. Discussion

The objectives of this study were to determine whether data generated by a commercially available leg-mounted tri-axial accelerometer can be used to identify play behaviour in calves up to 48 h old and to determine the optimal method for the analysis of the raw data generated to enable the occurrence of play behaviour to be accurately evaluated. The design of the study allowed us to assess different methods of analysing accelerometer-generated data without needing to repeat data collection, thus keeping animal usage to a minimum. The cumulative MI over 48 h correlated well with both the total duration of play behaviour and the number of play bouts in the same 48 h observation period. Whilst this is a rather crude method of behavioural analysis, it is straightforward to calculate, and the data are readily available. This method is a way of comparing the duration and number of bouts of play behaviour exhibited by different calves over longer periods of time without the requirement for time consuming visual observations. As such, this method allows for the analysis and comparison of large numbers of calves over longer periods of time where visual observations would be impractical, meaning it can easily be applied in the wider context of on-farm welfare assessment. The cumulative 48 h MI was identified in this study as the most appropriate sampling period for the analysis of play behaviour, as there was the least interference by other behaviours. It is possible that longer sampling periods may be even more appropriate, and further research is needed to identify the sampling period most suited to the use of the cumulative MI for comparing the duration and number of bouts of play behaviour between calves. Unexpectedly, we identified a positive correlation between the number of lying bouts and MI in some observation periods; as lying is a low-activity behaviour, a negative correlation was expected. It is likely that this is due to the definition of “lying bout” and method of recording lying bouts employed by the IceTag accelerometer. A lying bout is recorded as having occurred when the IceTag moves from vertical to horizonal and back to vertical again; this corresponds to the animal moving from standing to lying (this initiates the recording of a lying bout) before standing back up again (thus terminating the recording of the lying bout). Although lying itself is associated with a low MI, a lying bout is both initiated and terminated by a transition between lying and standing, which is recorded as movement by the IceTag and presented as an increase in the MI. Consequently, increased numbers of lying bouts are associated with increased numbers of posture transitions, resulting in a positive correlation between number of lying bouts and cumulative MI in some observation periods.

Pilot work had previously indicated that play behaviour could not be accurately defined by the motion index at a 1 s resolution: the MI at 1 min and 15 min resolutions showed greater potential for detecting the presence or absence of play behaviour. As these are longer duration sampling intervals, patterns of play behaviour could not be accurately defined using these MI resolutions because multiple behaviours could have occurred during the sampling interval. Rather, the number of sampling intervals in which the MI exceeded a defined cut point could be calculated in a method analogous to one-zero sampling (using visual observations) where the number of sampling intervals in which a defined behaviour is observed (in this, case play) is calculated [14]. One-zero sampling produces a single score for the required recording session that is expressed as a proportion of the total number of sample intervals during which the defined behaviour was observed and has previously been used by authors recording play, as it can capture sporadic, short-duration behaviours and is suited to capturing patterns of behaviour that are clustered [14,42,64]. Short sampling intervals are optimal when one-zero sampling is used, and this was reflected in our findings that the sensitivity and specificity of 1 min sampling intervals was more accurately repeatable than the sensitivity and specificity of 15 min sampling intervals. Motion index cut points of ≥ 2.5 for a 1 min resolution and ≥ 24.5 for a 15 min resolution were determined to have the optimum sensitivity and specificity for detecting the presence/absence of play behaviour in each sampling interval. For practical use, these would need to be rounded up to MI thresholds of ≥ 3 and ≥ 25 because the MI is reported in whole integers. These cut points consistently overestimated the one-zero sampling score obtained from visual observations, possibly because of the different methods used to calculate each metric. Sensitivity and specificity are diagnostic test characteristics (in this case, the “diagnostic test” is the selected MI cut point) and account for both positive and negative results (both true and false) [53], whereas one-zero sampling only records the number of positive samples out of the total number of recorded samples [14] and may therefore have included false positive results as well as true positive results. The optimised MI for detecting play behaviour was determined in this study based on a balance between sensitivity and specificity; the method was chosen because the presence of false negatives and presence of false positives were considered to have equal importance for our analysis. If attempting to replicate visual one-zero sampling, false negative results affect the calculated one-zero score less than false positive results (because only positive results contribute to the calculation of a one-zero score); therefore, a MI with higher specificity for indicating play behaviour is more suitable for this purpose despite the associated loss of sensitivity.

An unavoidable limitation of any study of the behaviour of neonatal calves is the large proportion of time calves of this age spend lying [55,65,66]. Play behaviour, in particular, is infrequent in calves of this age [55]; therefore, the absence of play predominates over the presence of play, which will have had an effect on the calculated sensitivity and specificity. In clinical medicine, the sensitivity and specificity of a test are typically used to calculate the positive and negative predictive value of the test—i.e., how good the test is at predicting the presence or absence of a specified condition in individuals [53]. Trénel et al. (2009) reported that the IceTag accelerometer had poor predictive value for movement [33]. Despite achieving high sensitivity and specificity, the optimised MI for detecting play behaviour in 1 min and 15 min intervals consistently overestimated the proportion of sampling intervals in our study, seemingly supporting the findings of Trénel et al. [33]. Positive and negative predictive value is affected by the prevalence of a condition in a population [53] (in this case, the duration of time spent engaged in play behaviour) and therefore would be different for each calf studied, as the unit of study was the individual calf and not the population. Consequently, positive and negative predictive value has limited value for this type of analysis, and the predicted value for selected MI cut points was not calculated in our study.

Classification and Regression Tree analysis is a methodology that is well suited to this type of study as it does not assume any particular data distribution and can tolerate imbalanced datasets [22,67]. In this study, we were interested in determining whether there was potential for a single MI threshold (cut point) to detect the presence of locomotor play in selected sample intervals; therefore, only the top nodes of the classification tree were of interest, and two-node trees were selected for both sample intervals. This approach is simple to interpret and has allowed us to identify single MI thresholds for both 15 min and 1 min sample intervals that have a high sensitivity and specificity for detecting play behaviour with acceptable accuracy. A more complex decision tree may allow play behaviour to be detected with even greater accuracy, and further work is warranted to develop predictive models for this purpose. However, increasingly complex decision trees are also increasingly difficult to interpret, and there is a risk that in the pursuit of increasing the accuracy of prediction, the models that are used become less applicable to the wider welfare and farming industries.

One-zero sampling can be limited in its application, as it does not always accurately reflect the true duration of a behaviour and is not a true reflection of the frequency of bouts of behaviour, as only the first bout observed in a sampling interval is recorded [15,54]. However, one-zero sampling is a practical method of recording the behaviour of large numbers of animals over longer periods of time, has good inter-observer reliability and is a suitable method for recording behaviour when only the presence or absence of a defined behaviour is of interest [54]. Recording visual observations using focal one-zero sampling is a well recognised technique in behavioural studies [14] and was well suited to matching the data output generated by the IceTag accelerometer at 1 min and 15 min resolutions; additionally, this study method was easily replicated in a different group of calves with good results. Although this method offers a good technique for comparing the frequency of positive sample intervals between calves, its accuracy is limited as it is not possible to be certain that positive visual and MI sample intervals are accurately matched using this method. As such, whilst this method is of value in situations where a simple count of positive samples is of interest, it cannot be used if the temporal pattern of calf play behaviour needs to be determined. This limitation can, however, be improved using longer sample intervals, which better compensate for the lag time in recording a change in velocity. Although the calculated sensitivity and specificity of selected MI cut points to indicate play were accurately repeated when the optimised MI for detecting play was applied to the video footage of a second group of calves, the proportion of sample intervals in which play occurred was consistently overestimated. We therefore consider that the calculation of the proportion of sample intervals in which play occurs (out of the total number of samples)—analogous to a one-zero sampling score—is a method preferable to the calculation of sensitivity and specificity for the ability of selected MI cut points to detect the presence/absence of play behaviour in each sampling interval.

Play behaviour is an indicator of positive animal welfare [48,51] and has potential for use in the assessment of the welfare of young calves, especially when comparisons between calves experiencing different challenges or environments are required. Traditional methods of recording behaviour in animals can be time consuming, laborious and not always suitable for on-farm welfare assessment. The value of accelerometer technology is already well recognised for monitoring behaviour in adult cattle and has benefits over traditional methods of recording behaviour; in particular, the ability to record activity over long periods of time [43] and generate large amounts of data that would be impractical to generate using visual observation techniques [31]. This study has described and evaluated different methods of utilising raw data generated by a commercially available tri-axial accelerometer to detect play behaviour in very young calves without the need for extra software or advanced data manipulation. The cumulative MI over 48 h correlates well with the total number of bouts of play behaviour and the duration of play behaviour observed during the same observation period and can be used to provide a simple comparison of the amount of play behaviour exhibited by different calves. The sensitivity and specificity of selected MI cut points for detecting the presence/absence of play behaviour in 1 min and 15 min sampling intervals was repeatable, but the MI cut points with optimal sensitivity and specificity consistently overestimated the proportion of sample intervals positive for play. This latter method, therefore, may have less value for future application to the analysis of play behaviour in very young calves; however, if this method is chosen for use by future researchers, 1 min sampling intervals are recommended over 15 min sampling intervals, as the sensitivity and specificity of the optimised MI for detecting play during 1 min intervals were more accurately reproduced when it was applied to a second group of calves.

Locomotor play was analysed in the study reported, as this type of play involves leg movement and could therefore be captured by the IceTag accelerometer; however, social play behaviour is also of interest when assessing positive animal welfare [68]. Social play in calves typically involves head movement [49], which has the potential to be captured using accelerometers worn on the head or neck. Accelerometer devices are rarely used to detect social behaviours [19], and to our knowledge, the use of accelerometer-generated data to detect social behaviours in calves has not yet been studied. Further work is warranted to determine whether accelerometers worn on the head or neck have potential for use as a tool for detecting calf social play behaviours.

The calves studied were all dairy or dairy x beef calves housed in group pens away from the dam in a rearing system typical of the UK dairy industry. Further work is needed to determine whether our findings can be extrapolated to calves housed and reared in different systems (e.g., in individual pens or in a beef suckler system) and whether similar findings are obtained when similar methods for analysis are applied to data generated by IceTag accelerometers worn by calves older than those recruited for this study.

## 5. Conclusions

In this study, cumulative accelerometer data generated over periods of time up to 48 h in duration correlated well with the duration of time engaged in play behaviour as well as the number of play bouts observed during the same observation period. Additionally, the MI recorded in 1 min and 15 min intervals is a good indicator of the presence/absence of play in each sampling interval and is analogous to a recognised method for recording behaviour. As play behaviour is considered to be an indicator of a positive welfare state, these findings suggest that the IceTag accelerometer has potential for future use as a tool in the assessment of the welfare of newborn dairy calves housed in group pens. The ease and speed of the reported methods of analysis for the identification of play behaviour in calves means that these methods have the potential to be practical options in the future for recording play behaviour in large numbers of calves and may be of particular value in situations where time is limited, such as in farm welfare audits. In such situations, the benefits gained by being able to rapidly analyse data from large numbers of calves may outweigh the detail missed using these methods instead of continuous visual observations, especially where the only behaviour of interest is locomotor play. Further work is needed to assess the use of these accelerometers in calves housed in different management systems, as well as in calves of different ages and types, before the use of this technology can be extended to the welfare assessment of all calves.

## Figures and Tables

**Table 1:** Ethogram of calf behaviour applied to continuous visual observations [49,55].

Behaviour	Behavioural Description	Key	Category
Lying	The calf is in a lying position. This includes both sternal and lateral recumbency. The head may be either elevated in an alert position or rested on the ground or any part of the body.	L	Resting
Standing	The calf is in an upright standing position, all legs are extended beneath the body, and all four feet are on the ground. The calf may be still or concurrently engaged in other active behaviours.	S	Active
Posture change	The calf is moving from standing to lying or lying to standing. Both hindlimbs are extended with feet on the ground, and one or both forelimbs are flexed at the carpus with the antebrachium in contact with the ground.	PC	
Step	Step activity associated with movement of the right rear leg.	ST	
Feeding	The calf is drinking milk.	F	
Head shake	The head is shaken, rotated or tossed (HS). Further defined by recording of concurrent forelimb (HFFM) or hindlimb (HRFM) movement if observed.	HS/HFFM/HRFM	Play
Hop	Upward movement of either the two forelimbs (HPF) or the two hindlimbs (HPR) in a vertical direction simultaneously.	HPF/HPR	
Leap forward	Both forelimbs are simultaneously lifted from the ground and stretched forward, causing the forequarters of the body to be lifted and the calf to move in a forward direction.	LF	
Leap sideward	All four limbs are elevated off the ground, and the calf moves in a lateral direction. All four feet land on the ground simultaneously.	LS	
Turn	Both forelimbs are lifted from the ground and stretched forward and laterally. The forequarters of the body are lifted, and the calf turns to one side. The direction of movement is upward, lateral and forward.	T	
Reverse	The calf moves in a backwards direction.	RV	
Running	Gait that is faster than a walk and contains a brief period of suspension.	R	
Buck low	Both hindfeet are simultaneously elevated to a level below the tarsus whilst both forelimbs remain in contact with the ground. The body lifts from front to back, and the head is lowered.	BL	
Buck high	Both hindfeet are simultaneously elevated to a level above the tarsus whilst both forelimbs remain in contact with the ground. The body lifts from front to back, and the head is lowered.	BH	
Buck kick	Both hindfeet are simultaneously elevated to a level equal to, or above, the tarsus, and one or both hindlimbs are kicked away from the body in a caudal or lateral direction. The body lifts from front to back, and the head is lowered.	BK	
Kick	One rear leg is kicked away from the body in a caudal or lateral direction. The other three limbs remain in contact with the ground.	K	
Management practice	Management practices performed by the farm staff that influence the calf’s behaviour and movement.	M	N/A
Out of View	The calf cannot be observed on the video footage.	O	

**Table 2:** Simplified ethogram of calf behaviours applied to one-zero sampling with a focus on the presence/absence of locomotor play behaviour in selected sample intervals.

Behaviour	Behavioural Description	Key	Behavioural Category
Play	Calf engages in locomotor play—defined as running (moving at a faster pace than walking with a period of elevation), jumping (all four limbs are lifted away from the ground at the same time—the calf may remain in the same position in space or may move forwards, backwards or laterally during the jump), bucking (both hindlimbs are elevated at the same time and kicked away from the body, either caudally or laterally—the calf may remain in the same plane of motion or may twist the body during the buck), kicking (one or both hindlimbs is elevated to a height above the tarsus and kicked out caudally or laterally from the body—the calf may be in motion or may remain still), hopping (the calf lifts both forelimbs away from the ground at the same time—the calf may remain in the same position in space, or the forequarters may move forwards, backwards or laterally during the hop), and spinning (calf lifts both hindlimbs at the same time and moves both around the central axis in either a clockwise or anticlockwise direction; the front of the body turns but remains in the same place in space, and both forelimbs remain on the ground).	p	Play
Lying	The calf is lying on the ground in any position. The whole body is in contact with the ground. The head may or may not be in contact with the ground.	l	Lying
Posture change	The calf is transitioning either from a lying to standing position or from a standing to lying position. The forelimbs are bent, and the antebrachium is in contact with the ground. The hindlimbs are straightening, and only the feet are in contact with the ground. The chest/sternum may be in contact with the ground. The abdomen and/or hindquarters do not contact the ground. The head may or may not be in contact with the ground.	s	Active (excl. play)
Standing	The calf is standing still with all four feet on the ground and all four limbs straight. No other part of the body is in contact with the ground.	t	Active (excl. play)
Walking	The calf takes two or more steps in a forward or backward direction. Three limbs are in contact with the ground at any one time and no part of the body is in contact with the ground.	w	Active (excl. play)
Sidestepping	The calf steps or stumbles one or both hind limbs laterally without moving the front limbs, or the calf moves the hindlimbs individually around the forelimb axis without moving its position in space	f	Active (excl. play)

**Table 3:** Correlation coefficient and p-values for Pearson and Spearman rank correlation of motion index and observed behaviour (duration(s) and number of bouts) for each 24 h observation period and the combined 48 h observation period. Bold font indicates significant results.

Behaviour	Observation Period				
	0 to 12 h	12 to 24 h	24 to 36 h	36 to 48 h	Combined 48 h
Lying (no. bouts)	0.277	0.317	**0.782**	0.448	**0.607**
	0.384	0.342	**0.008**	0.226	**0.048**
Lying (duration (s))	−0.035	0.027	−0.031	−0.309	0.264
	0.914	0.937	0.931	0.418	0.433
Play (no. bouts)	0.562	**0.811**	**0.926**	**0.871**	**0.922**
	0.057	**0.002**	**<0.001**	**0.002**	**<0.001**
Play (duration (s))	0.388	**0.829**	**0.918**	**0.937**	**0.773**
	0.213	**0.002**	**<0.001**	**<0.001**	**0.005**
Active excl. play (no. bouts)	0.250	0.175	**0.780**	0.215	0.552
	0.433	0.607	**0.008**	0.579	0.078
Active excl. play (duration (s))	0.124	0.273	**0.879**	**0.726**	0.386
	0.701	0.417	**0.001**	**0.027**	0.241
Active incl. play (no. bouts)	0.481	**0.726**	**0.995**	**0.835**	0.552
	0.114	**0.011**	**<0.001**	**0.005**	0.078
Active incl. play (duration (s))	0.144	0.273	**0.887**	**0.749**	0.386
	0.656	0.417	**0.001**	**0.020**	0.241

Upper row for each behaviour = correlation coefficient. Lower row for each behaviour = *p*-value.

**Table 4:** Sensitivity and specificity calculations for selected motion index cut points for 15 min sampling intervals (number of sample points = 396).

Motion Index * (15 min)	Sensitivity	Specificity	Balanced Accuracy
300	19.6%	100%	59.8%
200	29.4%	99.7%	64.6%
100	62.8%	98.6%	80.7%
50	84.3%	95.9%	90.1%
45	90.2%	95.4%	92.8%
40	90.2%	92.2%	91.2%
35	94.1%	92.2%	93.2%
30	96.1%	91.0%	93.6%
28	96.1%	90.4%	93.3%
27	96.1%	90.1%	93.1%
26	96.1%	90.1%	93.1%
25	98.0%	89.9%	93.6%
20	98.0%	87.8%	92.9%

* cut point set at greater than or equal to each selected motion index (MI). All values calculated manually for each cut point from all available data.

**Table 5:** Sensitivity and specificity calculations for selected motion index cut points for 1 min sampling intervals (number of sample points = 5938).

Motion Index * (1 min)	Sensitivity	Specificity	Balanced Accuracy
60	21.7%	99.8%	60.8%
50	30.3%	99.7%	65.0%
40	38.8%	99.5%	69.2%
30	54.0%	99.2%	76.6%
20	69.1%	98.5%	83.8%
10	90.1%	96.3%	93.2%
5	96.1%	94.3%	95.2%
3	98.0%	92.9%	95.5%

* cut point set greater than or equal to each selected MI. All values calculated manually for each cut point from all available data.

**Table 6:** Comparison of one-zero sampling score produced by visual observations (top row) and the equivalent score produced by motion index (MI) optimised for sensitivity/specificity (middle row), and that produced by MI optimised for one-zero sampling (lower row) for both 15 min and 1 min sampling intervals.

Calculation	Sample Interval Duration	Analysis	Sampling Method	Number of Positive Sample Intervals ^1^	Total Number of Sample Points	One-Zero Score ^2^
Initial calculation	1 min	CART	Visual observations	152	5938	0.026
			MI ≥ 2.5	560	5938	0.094
		Manual calculation	Visual observations	74	3501	0.021
			MI ≥ 23	76	3501	0.022
	15 min	CART	Visual observations	51	396	0.129
			MI ≥ 24.5	85	396	0.215
		Manual calculation	Visual observations	27	234	0.115
			MI ≥ 62	27	234	0.115
Results when applied to Dataset 2 *	1 min	Manual calculation	Visual observations	78	2437	0.032
			MI ≥ 23	95	2437	0.039
	15 min	Manual calculation	Visual observations	24	162	0.148
			MI ≥ 62	23	162	0.142

* One-zero sampling only (sensitivity and specificity validated using different methodology). ^1^ A positive sample interval was defined as a sample interval in which play was observed (visual one-zero sampling) or a sample interval in which the MI equaled or exceeded the selected cut point (IceTag equivalent sampling). ^2^ Number of positive sample points/total number of sample points. CART = Classification and Regression Tree analysis.

## References

[1] Ortega D.L., Wolf C.A. (2018). Demand for farm animal welfare and producer implications: Results from a field experiment in Michigan. Food Policy.

[2] von Keyserlingk M.A.G., Weary D.M. (2017). A 100-Year Review: Animal welfare in the Journal of Dairy Science—The first 100 years. J. Dairy Sci..

[3] Main D.C.J., Webster A.J.F., Green L.E. (2001). Animal Welfare Assessment in Farm Assurance Schemes. Acta Agric. Scand. Sect. A Anim. Sci..

[4] Main D.C.J., Mullan S., Atkinson C., Bond A., Cooper M., Fraser A., Browne W.J. (2012). Welfare outcomes assessment in laying hen farm assurance schemes. Anim. Welf..

[5] Main D.C.J., Whay H.R., Green L.E., Webster A.J.F. (2003). Effect of the RSPCA Freedom Food scheme on the welfare of dairy cattle. Vet. Rec..

[6] Veissier I., Butterworth A., Bock B., Roe E. (2008). European approaches to ensure good animal welfare. Appl. Anim. Behav. Sci..

[7] (2005). Farm Animal Welfare Council Report on the Welfare Implications of Farm Assurance Schemes. https://www.gov.uk/government/publications/fawc-report-on-the-welfare-implications-of-farm-assurance-schemes.

[8] Webster A.J.F., Main D.C.J., Whay H.R. (2004). Welfare assessment: Indices from clinical observation. Anim. Welf..

[9] Lawrence A.B., Vigors B., Sandøe P. (2019). What is so positive about positive animal welfare?—A critical review of the literature. Animals.

[10] Mellor D.J. (2015). Positive animal welfare states and encouraging environment-focused and animal-to-animal interactive behaviours. N. Z. Vet. J..

[11] Napolitano F., Knierim U., Grass F., De Rosa G. (2009). Positive indicators of cattle welfare and their applicability to on-farm protocols. Ital. J. Anim. Sci..

[12] Mellor D.J. (2012). Animal emotions, behaviour and the promotion of positive welfare states. N. Z. Vet. J..

[13] Barrell G.K. (2019). An Appraisal of Methods for Measuring Welfare of Grazing Ruminants. Front. Vet. Sci..

[14] Martin P., Bateson P. (2009). Recording Methods. Measuring Behaviour An Introductory Guide.

[15] Altmann J. (1974). Observational Study of Behavior: Sampling Methods. Behaviour.

[16] Mitlöhner F.M., Morrow-Tesch J.L., Wilson S.C., Dailey J.W., McGlone J.J. (2001). Behavioral sampling techniques for feedlot cattle. J. Anim. Sci..

[17] Chen J.M., Schütz K.E., Tucker C.B. (2016). Technical note: Comparison of instantaneous sampling and continuous observation of dairy cattle behavior in freestall housing. J. Dairy Sci..

[18] Hämäläinen W., Ruuska S., Kokkonen T., Orkola S., Mononen J. (2016). Measuring behaviour accurately with instantaneous sampling: A new tool for selecting appropriate sampling intervals. Appl. Anim. Behav. Sci..

[19] Brown D.D., Kays R., Wikelski M., Wilson R., Klimley A.P. (2013). Observing the unwatchable through acceleration logging of animal behavior. Anim. Biotelemetry.

[20] Rushen J., Chapinal N., De Passillé A.M. (2012). Automated monitoring of behavioural-based animal welfare indicators. Anim. Welf..

[21] Vasseur E., Rushen J., Haley D.B., de Passillé A.M. (2012). Sampling cows to assess lying time for on-farm animal welfare assessment. J. Dairy Sci..

[22] Ungar E.D., Nevo Y., Baram H., Arieli A. (2018). Evaluation of the IceTag leg sensor and its derivative models to predict behaviour, using beef cattle on rangeland. J. Neurosci. Methods.

[23] Müller R., Schrader L. (2003). A new method to measure behavioural activity levels in dairy cows. Appl. Anim. Behav. Sci..

[24] Neethirajan S. (2017). Recent advances in wearable sensors for animal health management. Sens. Bio-Sens. Res..

[25] Studd E.K., Boudreau M.R., Majchrzak Y.N., Menzies A.K., Peers M.J.L., Seguin J.L., Lavergne S.G., Boonstra R., Murray D.L., Boutin S. (2019). Use of Acceleration and Acoustics to Classify Behavior, Generate Time Budgets, and Evaluate Responses to Moonlight in Free-Ranging Snowshoe Hares. Front. Ecol. Evol..

[26] Barwick J., Lamb D.W., Dobos R., Welch M., Trotter M. (2018). Categorising sheep activity using a tri-axial accelerometer. Comput. Electron. Agric..

[27] Alvarenga F.A.P., Borges I., Palkovič L., Rodina J., Oddy V.H., Dobos R.C. (2016). Using a three-axis accelerometer to identify and classify sheep behaviour at pasture. Appl. Anim. Behav. Sci..

[28] Zobel G., Weary D.M., Leslie K., Chapinal N., von Keyserlingk M.A.G. (2014). Technical note: Validation of data loggers for recording lying behavior in dairy goats. J. Dairy Sci..

[29] Hammond T.T., Springthorpe D., Walsh R.E., Berg-Kirkpatrick T. (2016). Using accelerometers to remotely and automatically characterize behavior in small animals. J. Exp. Biol..

[30] Finney G., Gordon A., Scoley G., Morrison S.J. (2018). Validating the IceRobotics IceQube tri-axial accelerometer for measuring daily lying duration in dairy calves. Livest. Sci..

[31] Swartz T.H., McGilliard M.L., Petersson-Wolfe C.S. (2016). Technical note: The use of an accelerometer for measuring step activity and lying behaviors in dairy calves. J. Dairy Sci..

[32] Kok A., van Knegsel A.T.M., van Middelaar C.E., Hogeveen H., Kemp B., de Boer I.J.M. (2015). Technical note: Validation of sensor-recorded lying bouts in lactating dairy cows using a 2-sensor approach. J. Dairy Sci..

[33] Trénel P., Jensen M.B., Decker E.L., Skjøth F. (2009). Technical note: Quantifying and characterizing behavior in dairy calves using the IceTag automatic recording device. J. Dairy Sci..

[34] Nielsen P.P., Fontana I., Sloth K.H., Guarino M., Blokhuis H. (2018). Technical note: Validation and comparison of 2 commercially available activity loggers. J. Dairy Sci..

[35] Vázquez Diosdado J.A., Barker Z.E., Hodges H.R., Amory J.R., Croft D.P., Bell N.J., Codling E.A. (2015). Classification of behaviour in housed dairy cows using an accelerometer-based activity monitoring system. Anim. Biotelemetry.

[36] Alsaaod M., Kredel R., Hofer B., Steiner A. (2017). Technical note: Validation of a semi-automated software tool to determine gait-cycle variables in dairy cows. J. Dairy Sci..

[37] de Passillé A.M., Jensen M.B., Chapinal N., Rushen J. (2010). Technical note: Use of accelerometers to describe gait patterns in dairy calves. J. Dairy Sci..

[38] Kour H., Patison K.P., Corbet N.J., Swain D.L. (2018). Validation of accelerometer use to measure suckling behaviour in Northern Australian beef calves. Appl. Anim. Behav. Sci..

[39] Roland L., Schweinzer V., Kanz P., Sattlecker G., Kickinger F., Lidauer L., Berger A., Auer W., Mayer J., Sturm V. (2018). Technical note: Evaluation of a triaxial accelerometer for monitoring selected behaviors in dairy calves. J. Dairy Sci..

[40] Williams L.R., Moore S.T., Bishop-Hurley G.J., Swain D.L. (2020). A sensor-based solution to monitor grazing cattle drinking behaviour and water intake. Comput. Electron. Agric..

[41] Luu J., Johnsen J.F., de Passillé A.M., Rushen J. (2013). Which measures of acceleration best estimate the duration of locomotor play by dairy calves?. Appl. Anim. Behav. Sci..

[42] Größbacher V., Bučková K., Lawrence A.B., Špinka M., Winckler C. (2020). Discriminating spontaneous locomotor play of dairy calves using accelerometers. J. Dairy Sci..

[43] Poulopoulou I., Lambertz C., Gauly M. (2019). Are automated sensors a reliable tool to estimate behavioural activities in grazing beef cattle?. Appl. Anim. Behav. Sci..

[44] Ledgerwood D.N., Winckler C., Tucker C.B. (2010). Evaluation of data loggers, sampling intervals, and editing techniques for measuring the lying behavior of dairy cattle. J. Dairy Sci..

[45] Bonk S., Burfeind O., Suthar V.S., Heuwieser W. (2013). Technical note: Evaluation of data loggers for measuring lying behavior in dairy calves. J. Dairy Sci..

[46] Sutherland M.A., Worth G.M., Cameron C., Ross C.M., Rapp D. (2017). Health, physiology, and behavior of dairy calves reared on 4 different substrates. J. Dairy Sci..

[47] Rushen J., de Passillé A.M. (2012). Automated measurement of acceleration can detect effects of age, dehorning and weaning on locomotor play of calves. Appl. Anim. Behav. Sci..

[48] Held S.D.E., Špinka M. (2011). Animal play and animal welfare. Anim. Behav..

[49] Jensen M.B., Vestergaard K.S., Krohn C.C. (1998). Play behaviour in dairy calves kept in pens: The effect of social contact and space allowance. Appl. Anim. Behav. Sci..

[50] Boissy A., Manteuffel G., Jensen M.B., Moe R.O., Spruijt B., Keeling L.J., Winckler C., Forkman B., Dimitrov I., Langbein J. (2007). Assessment of positive emotions in animals to improve their welfare. Physiol. Behav..

[51] Brown S.M., Klaffenböck M., Nevison I.M., Lawrence A.B. (2015). Evidence for litter differences in play behaviour in pre-weaned pigs. Appl. Anim. Behav. Sci..

[52] Marcet Rius M., Cozzi A., Bienboire-Frosini C., Teruel E., Chabaud C., Monneret P., Leclercq J., Lafont-Lecuelle C., Pageat P. (2018). Selection of putative indicators of positive emotions triggered by object and social play in mini-pigs. Appl. Anim. Behav. Sci..

[53] Dohoo I., Martin W., Stryhn H. (2009). Screening and diagnostic tests. Veterinary Epidemiologic Research 2nd Edition.

[54] Lehner P.N. (1992). Sampling methods in behavior research. Poult. Sci..

[55] Gladden N., Ellis K., Martin J., Viora L., McKeegan D. (2019). A single dose of ketoprofen in the immediate postpartum period has the potential to improve dairy calf welfare in the first 48 h of life. Appl. Anim. Behav. Sci..

[56] Gladden N., McKeegan D., Viora L., Ellis K. (2018). Postpartum ketoprofen treatment does not alter stress biomarkers in cows and calves experiencing assisted and unassisted parturition: A randomised controlled trial. Vet. Rec..

[57] IceRobotics. https://www.icerobotics.com/researchers/#research-software.

[58] Friard O., Gamba M. (2016). BORIS: A free, versatile open-source event-logging software for video/audio coding and live observations. Methods Ecol. Evol..

[59] Brodersen K.H., Ong C.S., Stephan K.E., Buhmann J.M. (2010). The balanced accuracy and its posterior distribution. Proc. Int. Conf. Pattern Recognit..

[60] Breiman L., Friedman J.H., Olshen R.A., Stone C.J. (1984). Classification and Regression Trees.

[61] Loh W.Y. (2014). Fifty years of classification and regression trees. Int. Stat. Rev..

[62] Burman P. (1989). A Comparative Study of Ordinary Cross-Validation, v-Fold Cross-Validation and the Repeated Learning-Testing Methods. Biometrika.

[63] James G., Witten D., Hastie T., Tibshirani R. (2013). Cross-validation. An Introduction to Statistical Learning with Applications in R.

[64] Diamond J., Bond A.B. (2004). Social Play in Kaka (Nestor meridionalis) with Comparisons to Kea (Nestor notabilis). Behaviour.

[65] Chua B., Coenen E., van Delen J., Weary D.M. (2002). Effects of Pair Versus Individual Housing on the Behavior and Performance of Dairy Calves. J. Dairy Sci..

[66] Hill T.M., Bateman H.G., Aldrich J.M., Quigley J.D., Schlotterbeck R.L. (2013). Short communication: Intensive measurements of standing time of dairy calves housed in individual pens within a naturally ventilated, unheated nursery over different periods of the year. J. Dairy Sci..

[67] De’Ath G., Fabricius K.E. (2000). Classification and regression trees: A powerful yet simple technique for ecological data analysis. Ecology.

[68] Sutherland M., Worth G., Cameron C., Verbeek E. (2019). Effect of morphine administration on social and non-social play behaviour in calves. Animals.

